# Health Literacy as a Major Contributor to Health-Promoting Behaviors among Korean Teachers

**DOI:** 10.3390/ijerph18063304

**Published:** 2021-03-23

**Authors:** Eun Jung Bae, Ju Young Yoon

**Affiliations:** 1 College of Nursing, Seoul National University, Seoul 03080, Korea; ej810303@snu.ac.kr; 2 Research Institute of Nursing Science, Seoul National University, Seoul 03080, Korea; 3 Center for Human-Caring Nurse Leaders for the Future by Brain Korea 21 (BK 21) Four Project, College of Nursing, Seoul National University, Seoul 03080, Korea

**Keywords:** health literacy, health-promoting behaviors, teachers, school health

## Abstract

Teachers are not only subjects of school health efforts but also role models for students’ health behaviors; teachers’ health-promoting behaviors can induce students’ healthy behaviors with their positive health outcomes. This study was an examination of personal factors, situational factors, and health literacy as influences on teachers’ health-promoting behaviors. A hierarchical multiple regression analysis was implemented based on an integrated model of health literacy. The study results showed that health literacy was the strongest predictor of teachers’ health-promoting behaviors. In addition, school type and school culture were situational factors related to the interpersonal relations and stress management domains of the Health-Promoting Lifestyle Profile II scale. These findings could serve as foundational evidence for developing programs at the individual and organizational levels that enhance teachers’ health-promoting behaviors.

## 1. Introduction

Health-promoting behaviors refer to individual actions directed toward attaining a positive health outcome, such as optimal wellbeing and productive living [1]. As the disease burden of non-communicable diseases has become a significant public health concern, health-promoting behaviors have increased in importance for preventing and controlling disease effects. There is overwhelming evidence that healthy lifestyles reduce the risks for various chronic medical conditions [2,3,4,5] and mitigate adverse disease effects [4,6]. A prospective study showed a strong decrease in mortality risk associated with increasing numbers of positive health behaviors [7]. Health-promoting behaviors are crucial determinants of health and wellbeing that relate to quality of life [8,9] and life satisfaction [9].

Teachers’ health-promoting behaviors are important both because they lead to positive health outcomes for teachers themselves and because teachers who engage in these behaviors serve as examples for their students to develop healthy behaviors [10,11,12]. In the current school health paradigm [13], teachers are both subjects and resources of school health who should be supported in strengthening their health-promoting behaviors at school while they at the same time model behaviors that can encourage students’ health-promoting behaviors. Therefore, identifying predictors that promote teacher’s health-promoting behaviors and providing interventions based on them are essential in school health.

As a factor related to health-related behaviors, health literacy is a concept of growing importance. Health literacy is defined as “knowledge, motivation, and competencies to access, understand, appraise, and apply health information in order to make judgments and take decisions in everyday life concerning healthcare, disease prevention, and health promotion to maintain or improve quality of life during the life course” [14]. Health literacy is an asset for empowering people to promote their own health promotion, which involves knowledge, motivation, skills, and competence [14,15]. In this context, health literacy can act as a crucial personal competence to strengthen teachers’ health-promoting behaviors.

Previous researchers reported on relationships between general health literacy and health behaviors in general populations [16,17,18,19]. In studies based on national surveys, there was a positive association between health literacy and physical activity [16,17]. Similarly, people with higher health literacy were less likely to smoke and drink regularly and more likely to exercise [18]. Health literacy was also associated with a healthy diet and managing stress [19].

This relationship is also clearly presented in the integrated model of health literacy proposed by Sørensen et al. [14]. In the integrated model, health-promoting behaviors are direct consequences of health literacy, although the model accounts for multiple pathways of influence. For instance, in the integrated model, personal factors such as gender, income, and age, and situational factors such as social support and environmental factors influence health-promoting behaviors.

Some researchers have reported that demographic factors are related to teachers’ health behaviors [20,21], as are individual teachers’ roles within the school settings. For instance, homeroom teachers responsible for guiding their students’ health [22] and teachers of health-related subjects [23,24,25] can be considered to understand health behaviors better, and their high awareness of health behaviors is more likely to lead to action [24].

Situational factors should also be considered as predictors of teachers’ health-promoting behaviors. The type of school could be an important environmental factor related to teachers’ health promotion. Secondary schools have insufficient health promotion programs available to students compared with primary schools [26]. It is also reported that the performance of health-promoting schools is higher in elementary schools than in middle and high schools [27]. These differences in efforts and environment according to school type to promote students’ health can also affect teachers’ health-promoting behaviors.

School culture is also an important situational factor in health-promoting behaviors as a teacher’s social environment. The social environment affects health behavior by reducing or inducing stress [28,29]. School culture also reflects social support among school members [30], and social support is an important predictor of health behaviors [14].

Despite worldwide health literacy initiatives to improve healthy behaviors [31], few researchers have investigated the relationships between health literacy and teachers’ health-promoting behaviors. In addition, research that does focus on teachers’ health-promoting behaviors has highlighted personal characteristics [23,32,33], and little research has been conducted on social environments.

**Figure 1:**
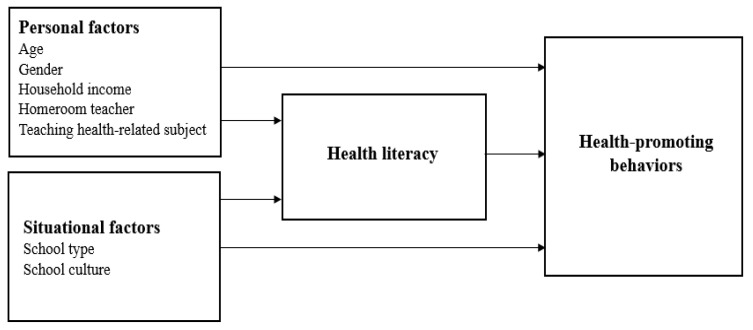
Conceptual framework of this study.

Therefore, this study aimed to identify personal factors, situational factors, and health literacy influences on teachers’ health-promoting behaviors based on an integrated model of health literacy. We also weighed how much each factor contributed to health-promoting behaviors. The conceptual framework of this study was derived from an integrated model of health literacy proposed by Sørensen et al. [14] and previous research evidence and is shown in Figure 1.

The research questions of this study were as follows: (1) What are the factors that influence teachers’ health-promoting behaviors?; (2) How much do personal factors, situational factors, and health literacy affect teachers’ health-promoting behaviors?

## 2. Methods

### 2.1. Data Collection and Samples

Participants were recruited using convenience sampling at elementary, middle, and high schools in Seoul, Incheon, and Gyeonggi-do regions where the districts were easily accessible for the researchers from September to October 2020. To obtain 95% power with significance of 0.05 and an expected medium effect size of 0.15 [34], a minimum sample size of 165 was required for hierarchical multiple regression analysis [35]. Researchers have indicated that sample sizes of 30–500 are appropriate for most studies and that 98% of the time, a sample of 500 assures that sample error will not exceed 10% of standard deviation [36,37]. We determined that we should recruit 500 participants based on these suggestions, after considering the researchers’ possible data collection. As of 2020, the teacher ratios by school type were 44%, 26%, and 30% for elementary, middle, and high schools, respectively; therefore, we planned to recruit 218, 130, and 152 teachers from each of the school types, respectively.

We conducted this survey online to comply with the educational institution’s COVID-19 guidelines related to social distancing. Using Google Forms, we produced three surveys according to the school type and designed them to end when the number of participants was satisfied. They were also designed to not proceed to the next question if the participant did not answer the current question. The recruitment documents and the online survey link were delivered through the school nurses’ network in three regions, and we asked the school nurses to share them with teachers through their schools’ online message boards. Participants read the purpose of the study before starting the online survey, including the guarantee that their participation would be voluntary and that they would be free to withdraw at any time. After we excluded 23 surveys with unreliable data (e.g., high school teachers’ responses on the elementary school survey) and three data outliers, we were left with 474 completed surveys for data analysis. This study was approved by the Seoul National University Institutional Review Board (IRB No. 2009/001-026).

### 2.2. Measures

#### 2.2.1. Personal Factors

The personal factors we measured were the teachers’ demographic characteristics (i.e., age, gender, household income) and whether they were currently homeroom teachers or teachers of health-related subjects, which included health, nutrition, and physical education.

#### 2.2.2. Situational Factors

The situational factors we measured were related to school type and culture. School type referred to whether the school was an elementary, a middle, or a high school, and we classified elementary schools as primary schools and middle and high schools as secondary schools. We measured school culture using 20 items of the school organizational culture subscale developed by Shin et al. [30], including items related to teachers’ relationships with administrators and colleagues (e.g., “Teachers at our school trust each other” and “The principal at our school understands the personal difficulties of teachers”). Respondents rated each item on a five-point scale ranging from 1 (strongly disagree) to 5 (strongly agree), and negative items were scored reversely, so that a higher score indicated a more positive school culture. Cronbach’s alpha was reported as 0.84 and 0.83, respectively, for teachers’ relationships with their administrators and colleagues [30], and we calculated an alpha of 0.840 for the combined subscale in the present study.

#### 2.2.3. Health Literacy

We measured teachers’ health literacy using 47 items of the European Health Literacy Survey Questionnaire (HLS-EU-Q 47) developed by the European Health Literacy Survey Consortium based on the integrated model of health literacy [17,38]. The previously translated and validated Korean version had already been used to measure health literacy in a Korean population [39]. HLS-EU-Q47 measures four competencies (access, understand, appraise, and apply information relevant to health) in three health-related domains (healthcare, disease prevention, and health promotion). Each item is rated on a four-point scale ranging from 1 (very difficult) to 4 (very easy). The HLS-EU-Q47 total scores were standardized to be between 0 and 50, following the (MEAN-1) × (50/3), and higher indices reflect higher health literacy. Researchers have reported Cronbach’s alphas ranging from 0.91 to 0.92 for the subscales and 0.97 for the total items [17]. For this study, we calculated Cronbach’s alphas that ranged from 0.917 to 0.952 for the subscales and 0.973 for the total items.

#### 2.2.4. Health-Promoting Behaviors

We measured teachers’ health-promoting behaviors using 52 items of the Health-Promoting Lifestyle Profile II (HPLP-II), developed by Walker et al. [40]. The previously translated and validated Korean version had been used to measure health-promoting behaviors [41]. HPLP-II consists of six domains: responsibility for health, nutrition, physical activity, stress management, interpersonal relations, and spiritual growth, and include items related to teachers’ current health-promoting behaviors (e.g., “Choose a diet low in fat, saturated fat, and cholesterol” and “Use specific methods to control my stress”). Each scale item is rated on a four-point scale ranging from 1 (never) to 4 (routinely). Higher scores indicate more health-promoting behaviors. Cronbach’s alpha coefficient has been reported to range from 0.793 to 0.872 for the subscales and was 0.943 for the total items [40]. Cronbach’s alpha ranged from 0.771 to 0.913 for the subscales and was 0.952 for the total items in the present study.

### 2.3. Data Analysis

Before performing formal analysis, we discarded any data outliers based on the standardized residual (d > 3) to minimize the disproportionate effect of the overall predictive ability of the model [42,43]. We used descriptive statistics to describe the characteristics of the study participants, calculating Pearson’s, point-biserial, and phi correlations according to combinations of scale types. We primarily used hierarchical regression to examine the predictors of health-promoting behavior. Hierarchical regression analysis is a sequential investigation of the influence of multiple predictors whereby the relative importance of a predictor is judged through incremental variance accounted by each predictor set [44]. We determined the order for entering variables into the model based on the theoretical framework [14] and logical considerations (Figure 1). In step 1, we entered the teachers’ personal factors (age, gender, monthly household income, homeroom teacher, health-related subject teacher); in step 2, we added situational factors (school type, school culture); and in step 3, we added health literacy. We also performed multiple regression analyses to identify factors that influenced the HPLP-II domains and evaluated the regression model assumptions [45]. A residual plot verified linearity and homoscedasticity. The normality was verified through the normal P–P plot of regression standardized residual. Multicollinearity was evaluated with the variance inflation factor (VIF). If the VIF value is greater than 4, it is assumed that multicollinearity exists. The analyses revealed no violations of the assumptions. We used IBM SPSS Statistics 24.0 (IBM Corp., Armonk, NY, USA) for the descriptive statistics and regression analysis and measured correlations using R version 3.3.0 (R Foundation for Statistical Computing, Vienna, Austria) for Windows.

## 3. Results

### 3.1. Sample Characteristics

The sample characteristics are shown in Table 1. Most of the participants were female (78.3%), and the mean age was 42.6 years; the participants’ mean monthly household income was 6.68 million KRW. By subject, 19.6% of the participants taught health-related subjects, and 51.9% were currently serving as homeroom teachers; by school type, 42.2% of the participants taught in elementary school. The mean overall school culture score was 70.33 (SD = 10.75). The mean score for general health literacy was 31.00 (SD = 7.59), and the means for healthcare health literacy, disease prevention health literacy, and health promotion health literacy were 30.87 (SD = 7.87), 31.44 (SD = 8.26), and 30.44 (SD = 8.78), respectively. The mean HPLP-II domain scores were lowest for physical activity and highest for interpersonal relations.

### 3.2. Correlation of Variables

The correlation coefficients among variables are presented in Table 2. We calculated three coefficients: Pearson’s for two continuous variables, point-biserial for one continuous and one binary variable, and phi for two binary variables. 

### 3.3. Regression Analysis

**Figure A1:**
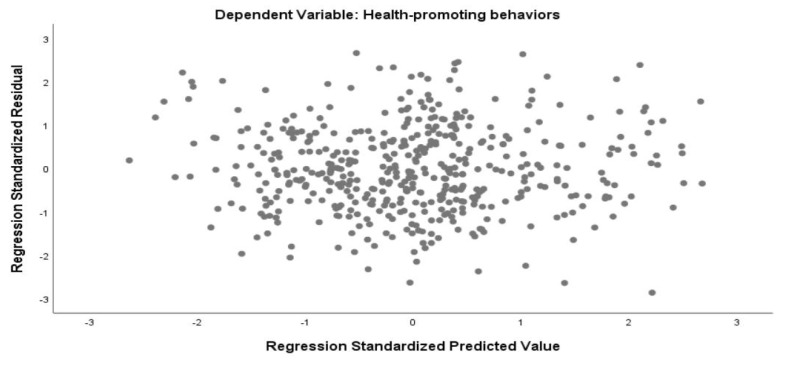
Scatter plot of standardized predicted value and standardized residual.

**Figure A2:**
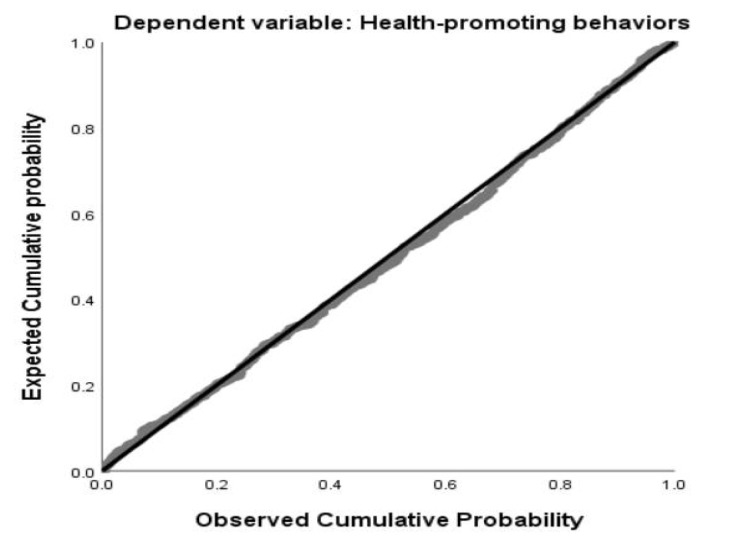
Normal P–P plot of regression standardized residual.

Figure A1 and Figure A2 in show the residual plot and normal P–P plot for testing assumptions of linear regression. Table 3 presents the hierarchical multiple regression results for predictors of health-promoting behaviors among teachers. In the first step, personal factors were entered as predictor variables, and these factors, as a whole, accounted for significant variance in health-promoting behaviors (F = 2.49, *p* = 0.026, R^2^ = 0.026 (adjusted R^2^ = 0.016)). The situational factors, as a whole, entered in the second step also explained significant variance in health-promoting behavior (Δ R^2^ = 0.044, Δ F = 10.988, *p* < 0.001, R^2^ = 0.070 (adjusted R^2^ = 0.056)). In the final step of the hierarchical regression analysis, health literacy full-scale scores contributed significant variance to health-promoting behaviors (Δ R^2^ = 0.238, Δ F = 25.89, *p* < 0.001, R^2^ = 0.308 (adjusted R^2^ = 0.296)), and in the final model, higher health literacy was associated with more health-promoting behaviors (β = 0.52, *p* < 0.001). Among the personal and situational factors, only school culture predicted health-promoting behaviors (β = 0.09, *p* = 0.021). In addition, in the final step, we entered each of the three sub-domains in health literacy, and all showed significant incremental variance (*p* < 0.001). Table 4 provides the results of the multiple regression analysis for factors affecting the HPLP-II subdomains. Health literacy was a significant predictor in all HPLP-II domains (*p* < 0.001). School type influenced nutrition (β = 0.10, *p* = 0.039) and stress management (β = 0.12, *p* = 0.008), and school culture predicted interpersonal relations (β = 0.14, *p* < 0.001) and stress management (β = 0.12, *p* = 0.005). In addition, Table A1, Table A2 and Table A3 demonstrate that all the HPLP-II sub-domains were significantly related to each domain of health literacy (i.e., healthcare, disease prevention, and health promotion).

## 4. Discussion

We examined personal factors, situational factors, and health literacy as influences on health-promoting behaviors among a population of Korean teachers based on an integrated model of health literacy. In particular, we focused on the relationship between health literacy and health-promoting behaviors. The significant contribution of this study is our finding that health literacy was a strong predictor of teachers’ health-promoting behaviors. It is also meaningful that we confirmed school organizations’ roles in encouraging health-promoting behaviors among teachers by examining the social environment as a determinant.

In this study, the mean score for Korean teachers’ health-promoting behaviors was not higher than previous HPLP-II findings for teachers [32,33]. In both the present and previous studies, respondents’ mean scores for HPLP-II nutrition, spiritual growth, and interpersonal relations domains were higher than the overall mean scores, and the means for health responsibility and physical activity were lower than the overall mean scores [32,33]. Schools are the best settings for encouraging children’s physical activity [46]. Although teachers are exposed to these environments, teachers’ physical activity may still be low because school policies to improve teachers’ health are lacking [47]. The lack of policies to improve teacher health may be due to teachers being viewed as resources that are not the proper subject of school health efforts [48]. To overcome these limitations, Poland, for example, has attempted a new project that includes promoting the health of teachers by expanding the existing student-centered health promotion activities. The investigators found that healthcare knowledge had increased among most of the staff following the intervention and that healthy behaviors among the staff had increased as well [47]. More efforts should be added for direct intervention to promote teachers’ health behaviors.

In this study, health literacy was significantly related to the teachers’ health-promoting behaviors, and these results aligned with previous study findings that health literacy was associated with healthy eating, physical activity, stress management, and reducing risky habits [16,17,18,19]. In particular, we confirmed with this study that health literacy was a strong predictor of health-promoting behaviors by demonstrating the most considerable change in incremental variance when we entered it in the final model. Health literacy had substantial influence on health-promoting behaviors even when other factors were controlled, and these findings give additional evidence for the importance of health literacy in public health [31].

We also investigated the relationships between health literacy and all HPLP-II domains. In a previous study conducted with health science students, only physical activity and health responsibility HPLP-II domains were related to health literacy [49], but in the current study, health literacy is positively associated with all sub-domains of HPLP-II. This finding suggests that improved health literacy can lead to a range of improvements in health status, including physical, mental, and spiritual health.

The strong predictability of health literacy on health-promoting behaviors identified in this study emphasizes the need to develop evidence-based interventions to enhance teachers’ health literacy. Recently, school nurses in Germany implemented interventions in 28 schools to improve teachers’ health literacy, and after the intervention, the prevalence of problematic or inadequate health literacy among teachers decreased from 49.9% to 45.8%, demonstrating the intervention’s effectiveness [50]. However, because all schools did not apply the prescribed curriculum equally, there was the limitation that the study authors could not conclusively determine the effectiveness of the curriculum’s content. Therefore, future studies are needed to identify effective interventions for improving teachers’ health literacy by developing and validating different curricula.

With this study, we verified that school culture as a situational factor influenced teachers’ health-promoting behaviors. Teachers who perceived positive relationships among school members showed higher scores on the HPLP-II interpersonal relations and stress management domains. School culture reflects a school’s social support networks among its members, and social support can influence better interpersonal and stress management behaviors by triggering positive coping strategies [51]. Previous studies have shown that teachers with more stress management behaviors had significantly fewer physical symptoms and reported higher teaching satisfaction [52]. Findings indicate that health-promoting behaviors in the social and emotional domains promoted by school culture can contribute to teachers’ wellbeing.

We found in this study that elementary school teachers had more stress management behaviors. This is in line with the previous study that Korean secondary school teachers experience higher job stress levels than elementary school teachers [53]. These results suggest that secondary school teachers need support to strengthen their stress management, and further investigation is needed to determine which school type factors affect teachers’ health-promoting behaviors. Such findings can contribute to developing and providing more specific organizational interventions.

This study has some limitations. First, the sampling was based on convenience, which limits the generalizability of our findings; further research is needed to ensure population representation through stratified sampling by region and school type. Second, this was a cross-sectional study; therefore, we could not establish temporality and causality; more longitudinal studies are required for exploring the causal relationships. Third, with the present study, we did not examine a broader range of environmental factors that can affect teachers’ health-promoting behavior. Additional factors such as the school’s physical environment and school health policies need to be investigated. Fourth, the current study was conducted during the coronavirus disease 2019 (COVID-19) pandemic. Uncertainty about COVID-19 prevention and management during the data collection period could influence teachers’ overall health literacy responses as one of the external factors; however, this study could not control the COVID-19 factor in the model. After the pandemic, further studies are needed to compare the differences of the role in health literacy to health-promoting behaviors, which could support the generalizability of this study based on an integrated model of health literacy.

## 5. Conclusions

In this study, health literacy was the strongest predictor of teachers’ health-promoting behaviors, and we consider improving health literacy an essential goal for enhancing schoolteachers’ health-promoting behaviors. School type and school culture were also significant in promoting healthy behaviors in the interpersonal relations and stress management domains of the HPLP-II. These results can contribute to developing interventions that improve school environments and encourage teachers’ health-promoting behaviors.

## Figures and Tables

**Table 1:** Characteristics of the participants (*N* = 474).

Variables	Category	N (%) or M ± SD
Gender	Male	103 (21.7)
	Female	371 (78.3)
Age		42.63 ± 9.24
	≤30	53 (11.2)
	31–40	148 (31.2)
	41–50	158 (33.3)
	≥51	115 (24.3)
Household income (monthly, 10,000 KRW)		668.82 ± 323.69
	≤ 50	82 (17.3)
	360–500	112 (23.6)
	510–700	107 (22.6)
	710–900	75 (15.8)
	≥910	98 (20.7)
Health-related subject teacher	Yes	93 (19.6)
	No	381 (80.4)
Homeroom teacher	Yes	246 (51.9)
	No	228 (48.1)
School type	Primary school	200 (42.2)
	Secondary school	274 (57.8)
School culture (20–100)		70.33 ± 10.75
General health literacy (index, 0–50)		31.00 ± 7.59
Healthcare health literacy (index, 0–50)		30.87 ± 7.87
Disease prevention health literacy (index, 0–50)		31.44 ± 8.26
Health promotion health literacy (index, 0–50)		30.40 ± 8.78
Health-promoting behaviors (52–208)		130.73 ± 22.81
Overall (1–4)		2.51 ± 0.43
Health responsibility (1–4)		2.27 ± 0.51
Physical activity (1–4)		2.22 ± 0.71
Nutrition (1–4)		2.57 ± 0.52
Spiritual growth (1–4)		2.74 ± 0.59
Interpersonal relations (1–4)		2.79 ± 0.52
Stress management (1–4)		2.45 ± 0.52

Note. M = mean; SD = standard error.

**Table 2:** Correlation of variables.

Variables	1	2	3	4	5	6	7	8	9
1. Gender	-								
2. Age	−0.02	-							
3. Household income	0.15	0.29	-						
4. Teaching health-related subject	0.07	0.15	0.05	-					
5. Homeroom teacher	−0.02	−0.25	−0.06	−0.41	-				
6. School type	0.30	−0.07	0.11	0.08	0.19	-			
7. School culture	−0.08	0.05	−0.04	0.01	0.04	0.06	-		
8. Health literacy	0.07	−0.09	0.12	0.16	0.004	0.18	0.17	-	
9. Health-promoting behaviors	0.05	−0.06	0.09	0.07	0.05	0.15	0.18	0.54	-

**Table 3:** Hierarchical multiple regression analysis predicting health-promoting behaviors among teachers.

Predictor	Model 1		Model 2		Model 3		Model 3(a)		Model 3(b)		Model 3(c)	
	*β*	*p*	*β*	*p*	*β*	*p*	*β*	*p*	*β*	*p*	*β*	*p*
Personal factors												
Age	−0.09	0.071	−0.10	0.056	−0.01	0.835	−0.01	0.788	−0.04	0.357	−0.01	0.775
Gender (ref. male)	0.03	0.565	0.01	0.788	0.004	0.917	−0.001	0.986	0.01	0.781	0.01	0.878
Household income ^1^	0.10	0.039	0.09	0.073	0.02	0.731	0.04	0.395	0.02	0.609	0.02	0.667
Teaching health-related subject	0.11	0.024	0.09	0.080	−0.004	0.933	0.01	0.852	−0.003	0.939	0.05	0.247
Homeroom teacher	0.08	0.130	0.04	0.481	0.03	0.535	0.03	0.578	0.03	0.466	0.03	0.545
Situational factors												
School type(ref. secondary school)			0.11	0.027	0.05	0.250	0.09	0.057	0.06	0.179	0.01	0.736
School culture			0.18	<0.001	0.09	0.021	0.11	0.010	0.11	0.007	0.10	0.012
Health literacy												
General HL					0.52	<0.001						
HC-HL							0.40	<0.001				
DP-HL									0.48	<0.001		
HP-HL											0.54	<0.001
*R* ^2^	0.026		0.070		0.308		0.211		0.282		0.335	
Adjusted *R*^2^	0.016		0.056		0.296		0.197		0.269		0.324	
*F (p)*	2.49 (0.030)		4.99 (<0.001)		25.89 (<0.001)		15.55 (<0.001)		22.78 (<0.001)		29.30 (<0.001)	
*Δ R* ^2^	0.026		0.044		0.238		0.141		0.212		0.265	

Note. ^1^ Log transformed; HL, health literacy; HC-HL, healthcare HL; DP-HL, disease prevention HL; HP-HL, health promotion HL; all coefficients are standardized.

**Table 4:** Multiple regression analysis predictors of health-promoting behaviors among teachers by Health-Promoting Lifestyle Profile II (HPLP-II) subdomain.

Predictor	Overall ^2^		Health Responsibility		Physical Activity		Nutrition		Spiritual Growth		Interpersonal Relations		Stress Management	
	*β*	*p*	*β*	*p*	*β*	*p*	*β*	*p*	*β*	*p*	*β*	*p*	*β*	*p*
Personal factors														
Age	−0.01	0.835	−0.07	0.118	0.05	0.354	0.20	<0.001	−0.01	0.801	−0.19	<0.001	−0.03	0.492
Gender (ref. male)	0.004	0.917	0.04	0.286	−0.10	0.026	0.12	0.009	−0.01	0.791	0.05	0.259	−0.05	0.244
Household income ^1^	0.02	0.731	−0.01	0.868	−0.05	0.290	0.04	0.366	0.05	0.336	0.06	0.194	−0.01	0.844
Teaching Health-related subject	−0.004	0.933	0.02	0.675	−0.002	0.965	0.02	0.624	0.01	0.890	−0.08	0.085	0.01	0.759
Homeroom teacher	0.03	0.535	0.01	0.795	0.07	0.142	0.03	0.532	−0.01	0.793	−0.01	0.885	0.03	0.530
Situational factors														
School type (ref. secondary school)	0.05	0.250	−0.002	0.959	0.07	0.131	0.10	0.039	−0.04	0.438	−0.01	0.915	0.12	0.008
School culture	0.09	0.021	0.04	0.296	0.03	0.475	0.04	0.308	0.07	0.119	0.14	<0.001	0.12	0.005
General HL	0.52	<0.001	0.52	<0.001	0.34	<0.001	0.36	<0.001	0.39	<0.001	0.45	<0.001	0.40	<0.001
*R* ^2^	0.308		0.298		0.140		0.220		0.165		0.297		0.228	
Adjusted *R*^2^	0.296		0.285		0.125		0.207		0.151		0.284		0.215	
*F (p)*	25.89 (<0.001)		24.62 (<0.001)		9.44 (<0.001)		16.42 (<0.001)		11.49 (<0.001)		24.50 (<0.001)		17.21 (<0.001)	

Note. ^1^ Log transformed; ^2^ This overall model is the same with model 3 in Table 3; HL, health literacy; all coefficients are standardized.

## Data Availability

The data presented in this study are available on request from the corresponding author. The data are not publicly available due to privacy.

## Appendix B

**Table A1:** Predictors of health-promoting behaviors among teachers by HPLP-II subdomain on the healthcare domain of health literacy.

Predictor	Overall ^2^		Health Responsibility		Physical Activity		Nutrition		Spiritual Growth		Interpersonal Relations		Stress Management	
	*β*	*p*	*β*	*p*	*β*	*p*	*β*	*p*	*β*	*p*	*β*	*p*	*β*	*p*
Personal factors														
Age	−0.01	0.778	−0.07	0.148	0.03	0.496	0.20	<0.001	−0.02	0.749	−0.19	<0.001	−0.04	0.444
Gender (ref. male)	−0.001	0.986	0.04	0.382	−0.10	0.028	0.11	0.013	−0.02	0.738	0.04	0.333	−0.05	0.231
Household income ^1^	0.04	0.395	0.01	0.762	−0.03	0.543	0.06	0.235	0.06	0.189	0.08	0.094	0.01	0.814
Teaching Health-related subject	0.01	0.852	0.03	0.582	0.01	0.787	0.03	0.571	0.02	0.738	−0.07	0.144	0.03	0.593
Homeroom teacher	0.03	0.578	0.01	0.836	0.07	0.154	0.03	0.560	-0.01	0.788	−0.01	0.868	0.03	0.555
Situational factors														
School type (ref. secondary school)	0.09	0.057	0.03	0.452	0.10	0.044	0.12	0.010	−0.01	0.864	0.03	0.540	0.15	0.001
School culture	0.11	0.010	0.05	0.192	0.05	0.274	0.05	0.232	0.08	0.069	0.16	<0.001	0.13	0.002
HC-HL	0.40	<0.001	0.43	<0.001	0.22	<0.001	0.30	<0.001	0.29	<0.001	0.36	<0.001	0.30	<0.001
*R* ^2^	0.211		0.219		0.081		0.185		0.109		0.230		0.165	
Adjusted *R*^2^	0.197		0.206		0.065		0.171		0.094		0.217		0.150	
*F (p)*	15.55 (<0.001)		16.31 (<0.001)		5.13 (<0.001)		13.20 (<0.001)		7.11 (<0.001)		17.39 (<0.001)		11.45 (<0.001)	

Note. ^1^ Log transformed; ^2^ This overall model is the same with model 3(a) in Table 3; HL, health literacy; HC-HL, healthcare HL; all coefficients are standardized.

**Table A2:** Predictors of health-promoting behaviors among teachers by HPLP-II subdomain on the disease prevention domain of health literacy.

Predictor	Overall ^2^		Health Responsibility		Physical Activity		Nutrition		Spiritual Growth		Interpersonal Relations		Stress Management	
	*β*	*p*	*β*	*p*	*β*	*p*	*β*	*p*	*β*	*p*	*β*	*p*	*β*	*p*
Personal factors														
Age	−0.04	0.357	−0.10	0.024	0.02	0.630	0.18	<0.001	-0.04	0.466	−0.21	<0.001	−0.06	0.213
Gender (ref. male)	0.01	0.781	0.05	0.222	−0.10	0.036	0.12	0.006	−0.01	0.886	0.05	0.204	−0.05	0.310
Household income ^1^	0.02	0.609	0.001	0.987	−0.04	0.355	0.05	0.322	0.05	0.291	0.06	0.157	−0.001	0.985
Teaching Health-related subject	−0.003	0.939	0.02	0.672	−0.001	0.991	0.02	0.649	0.01	0.909	−0.08	0.084	0.02	0.713
Homeroom teacher	0.03	0.466	0.02	0.708	0.08	0.127	0.03	0.484	−0.01	0.858	−0.002	0.969	0.03	0.479
Situational factors														
School type (ref. secondary school)	0.06	0.179	0.01	0.867	0.08	0.101	0.10	0.029	-0.03	0.522	0.003	0.950	0.13	0.005
School culture	0.11	0.007	0.06	0.144	0.04	0.322	0.05	0.204	0.08	0.068	0.16	<0.001	0.13	0.002
DP-HL	0.48	<0.001	0.48	<0.001	0.31	<0.001	0.34	<0.001	0.37	<0.001	0.43	<0.001	0.36	<0.001
*R* ^2^	0.282		0.269		0.124		0.213		0.155		0.282		0.202	
Adjusted *R*^2^	0.269		0.257		0.109		0.199		0.140		0.270		0.188	
*F (p)*	22.78 (<0.001)		21.42 (<0.001)		8.22 (<0.001)		15.73 (<0.001)		10.66 (<0.001)		22.85 (<0.001)		14.72 (<0.001)	

Note. ^1^ Log transformed; ^2^ This overall model is the same with model 3(b) in Table 3; HL, health literacy; DP-HL, disease prevention HL; all coefficients are standardized.

**Table A3:** Predictors of health-promoting behaviors among teachers by HPLP-II subdomain on the health promotion domain of health literacy.

Predictor	Overall ^2^		Health Responsibility		Physical Activity		Nutrition		Spiritual Growth		Interpersonal Relations		Stress Management	
	*β*	*p*	*β*	*p*	*β*	*p*	*β*	*p*	*β*	*p*	*β*	*p*	*β*	*p*
Personal Factors														
Age	−0.01	0.775	−0.08	0.081	0.05	0.262	0.19	<0.001	−0.02	0.743	−0.19	<0.001	−0.03	0.529
Gender (ref. male)	0.01	0.878	0.05	0.263	−0.10	0.022	0.12	0.008	-0.01	0.816	0.05	0.240	−0.05	0.239
Household income ^1^	0.02	0.667	0.001	0.995	−0.06	0.221	0.05	0.285	0.05	0.297	0.06	0.146	−0.01	0.794
Teaching Health-related subject	0.05	0.247	0.074	0.092	0.03	0.547	0.06	0.180	0.05	0.325	−0.28	0.524	0.05	0.237
Homeroom teacher	0.03	0.545	0.01	0.807	0.07	0.139	0.03	0.537	−0.01	0.780	−0.01	0.877	0.03	0.541
Situational Factors														
School type (ref. secondary school)	0.01	0.736	−0.03	0.464	0.04	0.413	0.08	0.094	−0.06	0.194	−0.03	0.492	0.09	0.052
School culture	0.10	0.012	0.05	0.184	0.03	0.531	0.05	0.204	0.07	0.087	0.15	<0.001	0.12	0.004
HP-HL	0.54	<0.001	0.51	<0.001	0.42	<0.001	0.33	<0.001	0.40	<0.001	0.44	<0.001	0.46	<0.001
*R* ^2^	0.335		0.295		0.195		0.206		0.175		0.292		0.276	
Adjusted *R*^2^	0.324		0.283		0.181		0.192		0.160		0.280		0.264	
*F (p)*	29.30 (<0.001)		24.31 (<0.001)		14.10 (<0.001)		15.068 (<0.001)		12.30 (<0.001)		24.02 (<0.001)		22.182	

Note. ^1^ Log transformed; ^2^ This overall model is the same with model 3(c) in Table 3; HL, health literacy; HP-HL, health promotion HL; all coefficients are standardized.

## References

[1] Pender N., Murdaugh C.L., Parsons M.A. (2011). Health Promotion in Nursing Practice.

[2] Warburton D.E., Bredin S.S. (2017). Health benefits of physical activity: A systematic review of current systematic reviews. Curr. Opin. Cardiol..

[3] Zhang X., Devlin H.M., Smith B., Imperatore G., Thomas W., Lobelo F., Ali M.K., Norris K., Gruss S., Bardenheier B. (2017). Effect of lifestyle interventions on cardiovascular risk factors among adults without impaired glucose tolerance or diabetes: A systematic review and meta-analysis. PLoS ONE.

[4] Schwingshackl L., Bogensberger B., Hoffmann G. (2018). Diet quality as assessed by the healthy eating index, alternate healthy eating index, dietary approaches to stop hypertension score, and health outcomes: An updated systematic review and meta-analysis of cohort studies. J. Acad. Nutr. Diet..

[5] Kyu H.H., Bachman V.F., Alexander L.T., Mumford J.E., Afshin A., Estep K., Veerman J.L., Delwiche K., Iannarone M.L., Moyer M.L. (2016). Physical activity and risk of breast cancer, colon cancer, diabetes, ischemic heart disease, and ischemic stroke events: Systematic review and dose-response meta-analysis for the Global Burden of Disease Study 2013. BMJ (Clin. Res. Ed.).

[6] Kelly J.T., Palmer S.C., Wai S.N., Ruospo M., Carrero J.J., Campbell K.L., Strippoli G.F. (2017). Healthy dietary patterns and risk of mortality and ESRD in CKD: A meta-analysis of cohort studies. Clin. J. Am. Soc. Nephrol..

[7] Khaw K.T., Wareham N., Bingham S.A., Welch A., Luben R., Day D. (2008). Combined impact of health behaviours and mortality in men and women: The EPIC-Norfolk prospective population study. PLoS Med..

[8] Mo P.K., Winnie W.M. (2010). The influence of health promoting practices on the quality of life of community adults in Hong Kong. Soc. Indic. Res..

[9] Lee M.K., Oh J. (2020). health-related quality of life in older adults: Its association with health literacy, self-efficacy, social support, and health-promoting behavior. Healthc. Multidiscip. Digit. Publ. Inst..

[10] Laguna M.C., Hecht A.A., Ponce J., Jue T., Brindis C.D., Patel A.I. (2020). Teachers as healthy beverage role models: Relationship of student and teacher beverage choices in elementary schools. J. Community Health.

[11] He L., Zhai Y., Engelgau M., Li W., Qian H., Si X., Gao X., Sereny M., Liang J., Zhu X. (2014). Association of children’s eating behaviors with parental education, and teachers’ health awareness, attitudes and behaviors: A national school-based survey in China. Eur. J. Public Health.

[12] Smuka I. (2012). Teacher role model and students’ physical activity. Pol. J. Sport Tour..

[13] Lewallen T.C., Hunt H., Potts-Datema W., Zaza S., Giles W. (2015). The whole school, whole community, whole child model: A new approach for improving educational attainment and healthy development for students. J. Sch. Health.

[14] Sørensen K., Van den Broucke S., Fullam J., Doyle G., Pelikan J., Slonska Z., Brand H. (2012). Health literacy and public health: A systematic review and integration of definitions and models. Bmc Public Health.

[15] Peerson A., Saunders M. (2009). Health literacy revisited: What do we mean and why does it matter?. Health Promot Int..

[16] Suka M., Odajima T., Okamoto M., Sumitani M., Igarashi A., Ishikawa H., Kusama M., Yamamoto M., Nakayama T., Sugimori H. (2015). Relationship between health literacy, health information access, health behavior, and health status in Japanese people. Patient Educ. Couns..

[17] (2012). HLS-EU Consortium. Comparative Report of Health Literacy in Eight EU Member States.

[18] Levin-Zamir D., Baron-Epel O.B., Cohen V., Elhayany A. (2016). The association of health literacy with health behavior, socioeconomic indicators, and self-assessed health from a national adult survey in Israel. J. Health Commun..

[19] Aygun O., Cerim S. (2020). The relationship between general health behaviors and general health literacy levels in the Turkish population. Health Promot. Int..

[20] Nagler E.M., Sinha D.N., Pednekar M.S., Stoddard A.M., Gupta P.C., Mathur N., Lando H., Aghi M., Shulman Cordeira L., Viswanath K. (2015). Social contextual factors and tobacco use among Indian teachers: Insights from the Bihar School Teachers’ Study. Prev. Med..

[21] Park H., Jung H.S., Lee J.H. (2009). Effects of Self-Efficacy on Health Promotion Lifestyle in Teachers. Korean Soc. Sch. Health.

[22] Lee J., Lee J. (2013). A survey on competencies of teachers of guidance using importance-performance analysis. J. Yeolin Educ..

[23] Lee J.H., Jung H.S., Choi E.S. (2003). Health promotion lifestyle profile of the teachers of health-related disciplines and not-health-related disciplines in middle and high school. Korean J. Occup. Health Nurs..

[24] Peltzer K., Pengpid S., Yung T.K., Aounallah-Skhiri H., Rehman R. (2016). Comparison of health risk behavior, awareness, and health benefit beliefs of health science and non-health science students: An international study. Nurs. Health Sci..

[25] Mikkonen K., Ojala T., Sjögren T., Piirainen A., Koskinen C., Koskinen M., Koivula M., Sormunen M., Saaranen T., Salminen L. (2018). Competence areas of health science teachers–A systematic review of quantitative studies. Nurse Educ. Today.

[26] Green E.T., Venta A. (2018). Lack of implementation of eating disorder education and prevention programs in high schools: Data from incoming college freshmen. Eat. Disord..

[27] Lee E.Y., Choi B.Y., Sohn A.R., Ahn D.H. (2009). Evaluating of health promoting school by school characteristics. Korean Soc. Health Educ. Promot..

[28] Institute of Medicine (2003). The Future of the Public’s Health in the 21st Century.

[29] McNeill L.H., Kreuter M.W., Subramanian S.V. (2006). Social environment and physical activity: A review of concepts and evidence. Soc. Sci. Med..

[30] Shin J.H., Y Y., Choi H.S. (2007). Predictive relations of teacher efficacy to school cultures. J. Child Educ..

[31] World Health Organization (2017). Shanghai declaration on promoting health in the 2030 Agenda for Sustainable Development. Health Promot. Int..

[32] Hong E., Kang Y.S., Ha Y. (2013). Factors affecting on health promoting behaviors among teachers with middle-aged women experiencing menopause. Korean J. Occup. Health Nurs..

[33] Jung S.H., Kim D.H. (2017). Health perception, health status, and health promoting behaviors of elementary school teacher. J. Korean Soc. Sch. Health.

[34] Cohen J., Hillsdale N.J. (1988). Statical Power Analysis for the Behavioral Sciences.

[35] Soper D. (2021). Hierarchical Multiple Regression Sample Size Calculator [Software]. https://www.danielsoper.com/statcalc.

[36] Roscoe J.T. (1975). Fundamental Research Statistics for the Behavioral Sciences.

[37] Hill R. (1998). What sample size is “enough” in internet survey research. Interpers. Comput. Technol. Electron. J. 21st Century.

[38] Sørensen K., Van den Broucke S., Pelikan J.M., Fullam J., Doyle G., Slonska Z., Kondilis B., Stoffels V., Osborne R.H., Brand H. (2013). Measuring health literacy in populations: Illuminating the design and development process of the European Health Literacy Survey Questionnaire (HLS-EU-Q). BMC Public Health.

[39] Kim J.H., Park C.Y., Kang S.H. (2019). A survey on the level and related factors of health literacy in Korean people. Health Policy Manag..

[40] Walker S.N., Hill-Polerecky D.M. (1996). Psychometric Evaluation of the Health-Promoting Lifestyle Profile II.

[41] Yun S.N., Kim J.H. (1999). Health-promoting behaviors of the women workers at the manufacturing industry - based on the Pender’s health promotion model. Korean J. Occup. Health Nurs..

[42] Sebert D.M., Montgomery D.C., Rollier D.A. (1998). A clustering algorithm for identifying multiple outliers in linear regression. Comput. Stat. Data Anal..

[43] Montgomery D.C., Peck E.A., Vining G.G. (2012). Introduction to Linear Regression Analysis.

[44] Petrocelli J.V. (2003). Hierarchical multiple regression in counseling research: Common problems and possible remedies. Meas. Eval. Couns. Dev..

[45] Hickey G.L., Kontopantelis E., Takkenberg J.J., Beyersdorf F. (2019). Statistical primer: Checking model assumptions with regression diagnostics. Interact. Cardiovasc. Thorac. Surg..

[46] Dobbins M., Husson H., DeCorby K., LaRocca R.L. (2013). School-based physical activity programs for promoting physical activity and fitness in children and adolescents aged 6 to 18. Cochrane Database Syst. Rev..

[47] Woynarowska-Sołdan M. (2019). Implementation trial of school staff health promotion: Polish experiences. Health Promot. Int..

[48] Shepherd J., Dewhirst S., Pickett K., Byrne J., Speller V., Grace M., Almond P., Hartwell D., Roderick P. (2013). Factors facilitating and constraining the delivery of effective teacher training to promote health and well-being in schools: A survey of current practice and systematic review. J. Public Health Res..

[49] Rueda-Medina B., Gómez-Urquiza J.L., Tapia-Haro R., Casas-Barragán A., Aguilar-Ferrándiz M.E., Correa-Rodríguez M. (2020). Assessing health science students’ health literacy and its association with health behaviours. Health Soc. Care Community.

[50] de Buhr E., Ewers M., Tannen A. (2020). Potentials of school nursing for strengthening the health literacy of children, parents and teachers. Int. J. Environ. Res. Public Health.

[51] Greenglass E.R. (1993). The contribution of social support to coping strategies. Appl. Psychol..

[52] Leung S.S., Wah Mak Y., Yu Chui Y., Chiang V.C., Lee A.C. (2009). Occupational stress, mental health status and stress management behaviors among secondary school teachers in Hong Kong. Health Educ. J..

[53] Lee H.H., Heo J., Kim J.M., Jung B.W., Oh S.A. (2017). A Study on the Actual Condition of Teacher Job Stress and Management Plan.

